# [Corrigendum] NEP1‑40 promotes myelin regeneration via upregulation of GAP‑43 and MAP‑2 expression after focal cerebral ischemia in rats

**DOI:** 10.3892/mmr.2023.12945

**Published:** 2023-01-23

**Authors:** Hong Zhao, Zhen-Dong Liu, Yong-Bo Zhang, Xiao-Yu Gao, Cui Wang, Yi Liu, Xun-Fen Wang

Mol Med Rep 24: 844, 2021; DOI: 10.3892/mmr.2021.12484

Subsequently to the publication of this paper, an interested reader drew to the authors’ attention that, in Fig. 4A on p. 6 showing the effects of NEP1-40 on MBP expression as determined via immunohistochemical analysis, certain of the data panels appeared to be overlapping, such that they may have been derived from the same original source. After having examined their original data, the authors have realized that these data panels were inadvertently assembled incorrectly.

A corrected version of Fig. 4 is shown below, incorporating data from one of the alternative experiments in Fig. 4A. Note that these errors did not significantly affect the results or the conclusions reported in this paper, and all the authors agree to this Corrigendum. The authors are grateful to the Editor of *Molecular Medicine Reports* for allowing them the opportunity to publish this Corrigendum, and apologize to the readership for any inconvenience caused.

## Figures and Tables

**Figure 4.**
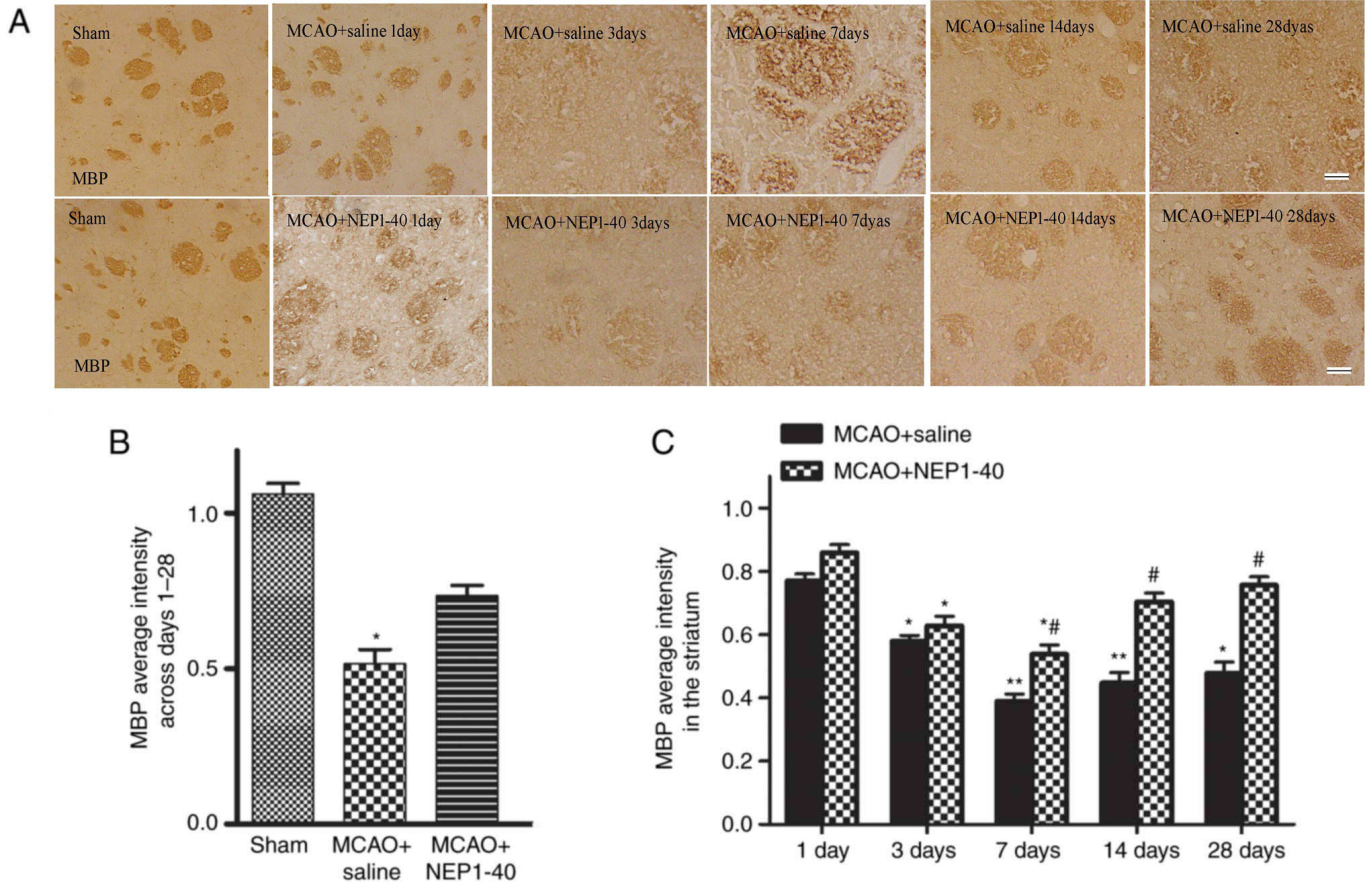
Effects of NEP1-40 on MBP expression as determined via immunohistochemistry. (A and B) Representative images of sections stained with anti-MBP antibody of the sham, MCAO + saline and MCAO + NEP1-40 groups at days 1, 3, 7, 14 and 28 after MCAO (scale bar, 50 µm). (C) Quantification of MBP intensity. MBP immunoreactivity in the ipsilateral striatum was decreased after MCAO from day 3 to day 28, which was notably restored by NEP1-40 treatment. *P<0.05 and **P<0.01 vs. sham or day 1; ^#^P<0.05 vs. MCAO + saline. MCAO, middle cerebral artery occlusion; NEP1-40, Nogo extracellular peptide 1–40; MBP, myelin basic protein.

